# Identification of cultural determinants of antibiotic use cited in primary care in Europe: a mixed research synthesis study of integrated design “Culture is all around us”

**DOI:** 10.1186/s12889-015-2254-8

**Published:** 2015-09-17

**Authors:** Pia Touboul-Lundgren, Siri Jensen, Johann Drai, Morten Lindbæk

**Affiliations:** Department of Public Health, Archet 1 hospital, 1st level, Nice University Hospital, 151, rte St Antoine de Ginestière, CS 23079, 06202 Nice Cedex 3, France; Department of teaching and research in General Practice, Medical School, University of Nice – Sophia Antipolis, 28, avenue de Valombrose, 06107 Nice, France; Department of General Practice/Family Medicine, University of Oslo, Oslo, Norway; Antibiotic Centre for Primary Care, Institute of Health and Society, University of Oslo, Oslo, Norway

## Abstract

**Background:**

Inappropriate antibiotic prescribing, particularly for respiratory tract infections (RTI) in ambulatory care, has become a worldwide public health threat due to resulting antibiotic resistance. In spite of various interventions and campaigns, wide variations in antibiotic use persist between European countries. Cultural determinants are often referred to as a potential cause, but are rarely defined. To our knowledge, so far no systematic literature review has focused on cultural determinants of antibiotic use. The aim of this study was to identify cultural determinants, on a country-specific level in ambulatory care in Europe, and to describe the influence of culture on antibiotic use, using a framework of cultural dimensions.

**Method:**

A computer-based systematic literature review was conducted by two research teams, in France and in Norway. Eligible publications included studies exploring antibiotic use in primary care in at least two European countries based on primary study results, featuring a description of cultural determinants, and published between 1997 and 2015. Quality assessment was conducted independently by two researchers, one in each team, using appropriate checklists according to study design. Each included paper was characterized according to method, countries involved, sampling and main results, and cultural determinants mentioned in each selected paper were extracted, described and categorized. Finally, the influence of Hofstede’s cultural dimensions associated with antibiotic consumption within a primary care setting was described.

**Results:**

Among 24 eligible papers, 11 were rejected according to exclusion criteria. Overall, 13 papers meeting the quality assessment criteria were included, of which 11 used quantitative methods and two qualitative or mixed methods. The study participants were patients (nine studies) and general practitioners (two studies). This literature review identified various cultural determinants either patient-related (illness perception/behaviour, health-seeking behaviour, previous experience, antibiotic awareness, drug perception, diagnosis labelling, work ethos, perception of practitioner) or practitioner-related (RTI management, initial training, antibiotic awareness, legal issues, practice context) or both (antibiotic awareness).

**Discussion and Conclusion:**

Cultural factors should be considered as exerting an ubiquitous influence on all the consecutive stages of the disease process and seem closely linked to education. Interactions between determinant categories, cultural dimensions and antibiotic use in primary care are multiple, complex and require further investigation within overlapping disciplines. The context of European projects seems particularly relevant.

**Electronic supplementary material:**

The online version of this article (doi:10.1186/s12889-015-2254-8) contains supplementary material, which is available to authorized users.

## Background

Increasing antibiotic resistance due to inappropriate antibiotic prescription is a worldwide public health threat [1] which, according to the first WHO report in 2014 on the subject, could lead to a post-antibiotic era during the 21^st^ century [2]. The highest rates of antibiotic prescriptions are observed in primary care for respiratory tract infections (RTI) [1]. A large number of international and national studies have explored various aspects of this situation. In addition, campaigns targeting the general public, aiming to raise awareness of appropriate antibiotic use and of the dangers linked to antimicrobial resistance, have been carried out in several countries, as well as interventions targeting practitioners and other health professionals, with varying results [3]. In spite of all these initiatives, the differences in surveyed antibiotic consumption [4] and related antimicrobial resistance rates [5] between European countries remain considerable and persistent over the years.

The multiple reasons for these differences are not fully understood. Various determinants of antibiotic prescription have been suggested in the literature and cultural factors are often referred to as possible explanations of persistent differences in antibiotic consumption between countries. Harbarth and Monnet [6] published an overview of determinants that could explain differences in antibiotic use, which included cultural factors. They also highlighted the importance of conducting further research, in particular to clarify the influence of cultural and socioeconomic determinants to guide interventions targeting appropriate antibiotic use.

According to the MeSH thesaurus culture is defined as a collective expression for all behaviour patterns acquired and socially transmitted through symbols. Cultural dimensions have been identified by different authors such as Hofstede [7], Hampden-Turner, Trompenaars [8] and Schwartz [9] as a model to better understand and measure cultural differences using various developed tools [10]. Various tools have been developed to measure and quantify cultural dimensions. One of the most popular approaches is based on the extensive work conducted by Geert Hofstede a Dutch sociologist. He defined and described initially four different cultural dimensions [11]: Power distance (PD), which measures the way society treats inequality between individuals, Uncertainty avoidance (UA), measuring the way the society deals with the uncertainty linked to the future, Individualism versus collectivism (IC), which measures the link between an individual and his/her group, and Masculinity versus femininity (MF), which measures how gender roles are distributed in a society. A database was created where scores for each country and each cultural dimension could be quantified and compared between countries [11] as well as correlated to other variables such as antibiotic consumption [12, 13].

Different levels of cultural identity can be considered within a society (family, neighbourhood, school, youth groups, workplace, community etc.). A focus on country-specific culture in Europe appeared the most relevant for this study, considering the fact that interventions, campaigns, surveillance of antibiotic consumption and resistance as well as numerous European projects tackling the problem of bacterial resistance have all been conducted on a national scale within the European context.

Even though cultural factors are often referred to in the literature as one of the possible explanations for persisting differences in antibiotic consumption between countries, they are rarely defined and described. Furthermore, to our knowledge, there is no systematic literature review specifically describing cultural determinants of antibiotic use. The aim of this study was to identify and describe determinants of antibiotic use that are referred to in the literature as cultural, on a country-specific level in ambulatory care in Europe, and to better understand the influence of Hofstede’s cultural dimensions in a primary care setting in order to direct future research.

## Materials and method

### Search strategy

A computer-based mixed research synthesis study of integrated design was chosen as relevant to the final assimilation of results from both qualitative and quantitative findings [14]. Two research teams, one in France and one in Norway, independently conducted a literature search according to the same predetermined inclusion and exclusion criteria. The research strategy was devised with advice from an experienced librarian and included both medical and non-medical search engines: Pubmed, Cochrane Library, Science Direct, CAIRN, Erudit, Francis, http://www.openedition.org/, Wiley library online for the Journal of the Royal Anthropological Institute, PsycNet and Social science citation index.

Key words identified with the MeSH thesaurus included “culture” (MeSH definition described in the introduction section), “antibiotics”/“antibacterial agents”/“antimicrobial agents”. The computer-based search was completed by backward snowballing from reference lists of eligible articles. Papers in both English and French were included.

### Eligibility criteria

The following inclusion criteria were applied: cross-cultural studies concerning antibiotic use in primary care in at least two European countries which included a description of cultural determinants, published between 1997 and 2015, studies based on primary study results. Research focusing only on countries outside Europe or on a single European country, involving neither primary care, antibiotic use, nor cultural determinants, published before 1997 or after 2015 and which were not based on primary study results was excluded.

#### Study selection

The first exclusion was done manually after viewing the title or the abstract. Exclusion of eligible full text papers was based on exclusion criteria and quality assessment.

#### Study designs

Our literature review addressed both quantitative and qualitative studies. The study designs of the included quantitative studies were identified according to the algorithm suggested by the National Institute for Health and Clinical Excellence (NICE) 2012 [15] as correlation studies, and/or cross-sectional observational studies.

### Quality assessment

The quality assessment strategy (QA) included selection and adaptation of QA tools according to the identified study design(s) and an independent quality assessment by 2 researchers, one in each research team. QA tools were selected taking into account recommendations from a recent systematic literature review by Sanderson *et al.* [16] in 2007 concerning quality assessing tools in observational studies. The authors advised simple checklists rather than scales, having shown evidence of validity and reliability, including a small number of key domains, and being as specific as possible with regard to study design and topic area to avoid bias in quality assessment. The simple checklists mentioned in this literature review that applied to cross-sectional studies were however specific to other topic areas such as mental health. Thus, the following tools were used according to the defined criteria [16]: the NICE recommendation tools for both the correlation and qualitative studies (score 2+: All or most of the checklist criteria have been fulfilled, where they have not been fulfilled, the conclusions are very unlikely to alter/ + / -); the Effective Public Health Practice project (EPHPP) QA tool for quantitative studies for the cross-sectional observational studies (score 1: strong / 2: moderate / 3: weak) was used [17]. However, minor adaptations were made for non-applicable sections. Any differences between the two researchers were discussed to reach consensus, the lower score was used if differences persisted.

### Data extraction and description

Selected papers were then compiled and summarized using Zotero© software. To characterise each paper, the method, investigated countries, sampling and main results were extracted and tabulated in chronological order, initially according to their methodology, including the overall QA score.

Cultural determinants cited in both qualitative and quantitative selected papers were then extracted, categorized and tabulated including relevant references in a final unique table assimilating the results.

Finally, the influence of Hofstede’s cultural dimensions having shown an association with antibiotic consumption within the medical consultation in a primary care setting was identified and described.

## Results

### Article selection and characteristics of selected articles

Twenty-four eligible papers were identified, eight through free access search engines and 16 through backward snowballing from reference lists of eligible articles 11 of which were rejected according to exclusion criteria (six focused on a single country, two included countries outside Europe and three were not based on primary study results). In all, 13 papers that also met the QA assessment criteria (Additional file 1), taking into account their study design according to the applicable checklist scorings, were included in the literature review. The overall assessment score was two for cross-sectional studies with the adapted EPHPP tool and 2+ for correlation studies (internal and external validity) as well as for qualitative studies using the appropriate NICE checklists.

The characteristics of each selected paper were displayed in two separate tables according to their methodology: 11 studies used quantitative methods [12, 13, 18–26] (Additional file 2) and two studies used qualitative or mixed methods [27, 28] (Additional file 3).

Each study involved between two and 27 European countries, only one paper involved other international countries in addition to European ones. The Netherlands and Belgium were the most frequently studied. In quantitative studies, patient numbers ranged from 678 to 26 259.

### Culture-related results

Within the quantitative papers, patients’ knowledge, attitudes, behaviour and education towards antibiotic use and antibiotic resistance in the context of RTI were extensively explored [18–23, 25, 26] and were described as varying according to countries, in contrast to clinical outcomes. Different “patient types” were described in two studies [19, 20] according to treatment compliance and attitudes regarding antibiotics as well as respect or attitude towards the practitioner. These “patient types” were unequally distributed among countries. Geographical variation of appropriate attitudes according to location of the country in Europe (North versus South, East versus West) was observed in one study [23] which also found that awareness of antibiotic resistance was the main difference between the participating countries.

Only one paper focused on practitioners’ attitudes and beliefs [24] in 2 contrasting countries with regard to antibiotic use (France and the Netherlands), suggesting that cultural differences in patients’ health seeking behaviour and perception regarding medication could account, at least in part, for differences in antibiotic prescription patterns.

Two papers report a correlation between several of Hofstede’s cultural dimensions and European data concerning antibiotic consumption, and also patients’ knowledge of antibiotics [12, 13]; these correlations were found between high antibiotic consumption and high scores of PD [12], UA [12, 13] and MF [12, 13] but did not persist when controlling for wealth.

One of the qualitative studies explored patients’ attitudes, knowledge and behaviour towards RTI and antibiotics [27] in two similar towns in two countries with contrasting antibiotic use (Belgium and the Netherlands). Major differences in disease labelling and initial coping behaviour were described in addition to attitudes towards antibiotics and expectations. The other qualitative study explored GPs’ antibiotic prescription patterns [28] in France and the Netherlands and described differences in prescription contexts in each country in particular with regards to initial medical training, legal complaints, retribution system and practice context.

Among the selected studies, 11 targeted adult patients and two targeted GPs.

### Overview of categories of identified cultural determinants

The cited cultural determinants in each selected paper were extracted, categorized with regards to the consecutive stages of the disease process and described (Table 1) with relevant references. These determinants were patient-related (e.g. illness perception/behaviour and health-seeking behaviour, individual experience, drug perception, diagnostic label, work ethos, practitioner perception), practitioner-related (e.g. RTI management, initial training, legal complaints, practice context) or both (antibiotic awareness). Patients’ attitudes, beliefs and knowledge towards infections and antibiotics were the most frequently explored and identified cultural determinants.

**Table 1:** Overview of categories of identified cultural determinants

Cultural determinant	Description	References
Patient related determinants		
Illness perception/behaviour and health-seeking behaviour	Attitudes, knowledge and beliefs towards URTI symptoms (serious or self-limiting, belief in the healing power of the body, fear of complications), initial coping strategies, threshold for consulting a GP, in particular for self-limiting diseases.	[18, 21, 24, 25, 27]
Individual experience	Previous experience of similar episodes.	[27]
Antibiotic awareness	Attitudes, knowledge, beliefs and perceptions towards antibiotics (their effectiveness in speeding recovery and preventing complications, their adverse effects, antibiotic resistance).	[13, 19, 21, 23, 25–27]
Drug perception	Perception towards antibiotics and symptomatic medication: scepticism towards medications and fear of toxicity, or considered as accelerators of the healing process with fear of complications if no medicines were used.	[24, 27]
Labelling of diagnosis	Perception of what is considered as a real symptom and use of labels.	[27]
Work ethos	Behaviour towards work: continue working in spite of illness or stop working to let the body recover and avoid transmitting infection to others.	[13, 27, 28]
Practitioner perception	Perception of their practitioner’s competence, trust in the practitioner.	[12, 19, 20]
Practitioner related determinants		
RTI management	Attitudes towards RTI, management, including decision-making.	[12, 24, 28]
Initial training	Orientation of initial medical training (hospital-centred or outpatient-centred).	[28]
Antibiotic awareness	Attitudes towards and beliefs concerning antibiotics.	[12, 24]
Legal complaints	Antibiotic prescription to avoid legal complaints.	[28]
Practice context	Perceived patients’ expectations, patient education strategies, prescription patterns.	[12, 28]

### Relation between identified cultural determinants and Hofstede’s cultural dimensions in a primary care setting concerning antibiotics

The framework developed by Hofstede’s is the most frequently used in studies mentioning cultural influence on antibiotic use. According to several authors, the cultural dimensions of PD [12], UA [12, 13] and MF [12, 13] are correlated to antibiotic use. When these dimensions score highly, antibiotic prescription is seen as a sign of power and expertise (PD, Table 2), decreases GPs’ and patients’ fear linked to uncertainty of diagnosis and illness outcome (UA, Table 3) and is regarded as meeting the priority of continuing to work in spite of illness (MF, Table 4). The influence of these 3 cultural dimensions on the relationship and communication between the practitioner and the patient, which are closely linked to the decision to prescribe or not, is also reported (Tables 2–4). UA (Table 3) was the most frequently quoted cultural dimension from both the practitioners’ and the patients’ perspective.

**Table 2:** Influence of power distance within the medical consultation in primary care

High	Low	References
Patients look up to the GP.	Patients see themselves as equal to the GP.	[12]
Less shared decision making: “Doctor knows best” attitude (Less discussion, information, counselling and negotiation during the consultation).	More shared decision making (more discussions, information, counselling and negotiation in the consultation).	[12, 20, 28]
GP cannot acknowledge he is unsure of diagnosis (fear of inspiring less confidence).	GP can acknowledge he is unsure of diagnosis (inspires confidence anyway).	[12]
Antibiotic prescription symbolic sign of power and expertise.	Antibiotic has a less symbolic importance.	[12]

**Table 3:** Influence of uncertainty avoidance within the medical consultation in primary care

High	Low	References
Patients have high risk perception of the threat of the disease and possible complication.	Patients have low risk-perception of the threat of the disease and of possible complications.	[19, 21, 27]
Patients feel confident only if they have a disease with a clear cause, label and treatment (defensive medicine).	Patients feel confident even in case of uncertainty, accepting that the GP has no specific diagnosis or that no treatment can be given.	[12]
Patients prefer a “rather safe than sorry” attitude.	Patients accept a “wait and see” attitude.	[12, 19, 27]
The illness is perceived as an evil phenomenon against which you should fight.	The illness is perceived as a natural phenomenon with a natural history to be respected.	[24]
GPs feel uncomfortable and are anxious of making mistakes.	GPs are aware of the dangers of a defensive attitude.	[12]
GPs see themselves as experts and feel the inner urge to “do something”; prescribe what’s considered to be the less risky for the patient on a short term basis.	GPs accept a degree of uncertainty and a “wait and see” approach.	[12]
Prescribing antibiotics decreases the fear linked to uncertainty of both the GP and the patient.	Prescribing antibiotics does not decrease uncertainty-related fear of the GP nor of the patient.	[12, 13, 19, 27]

**Table 4:** Influence of Masculinity feminity within the medical consultation in primary care

Masculine societies	Feminine societies	References
The patient should not be ill, the patient needs to return to work/activity very quickly.	The patient can be ill and this can excuse absence from work/activity	[13]
Antibiotics are regarded as a vital medicine to get back to work as quickly as possible which is felt as a priority.	Antibiotics are not regarded as a vital medicine and getting back to work as quickly as possible is not felt as a priority.	[13]

## Discussion

### Main findings: “Culture is all around us”

The findings of this literature review allowed us to identify and categorize cultural determinants (Table 1) in different cultural primary care settings and to confront them with Hofstede’s cultural dimensions (Tables 2–4). The different determinants described as cultural in the selected papers concern the various stages of the disease process from the patient’s illness perception and health seeking behaviour up to the decision-making stage at the end of the consultation, suggesting an ubiquitous influence of culture. Several narrative literature reviews describe determinants of antibiotic consumption, identifying cultural determinants as one particular category among others, which are often described as linked to the practitioner, the patient, the health care system, the pathogen’s characteristics etc. [6, 29–32]. Thus, our literature review highlights the extensive influence of culture at different levels rather than being a category among other determinants. This is confirmed by Monnet’s and Harbarth’s conclusion that cultural and socioeconomic factors pervade all aspects of antibiotic use [6].

### Strengths and limitations

To our knowledge this is the first systematic review identifying and describing cultural determinants. The method we chose, i.e. to initially classify papers according to their methodology made it easier for us to identify future research in this field. The lack of exclusively qualitative studies was striking. Only two qualitative papers were identified, one involving patients and another involving GPs. Within the quantitative papers, patients’ knowledge, attitude and education concerning antibiotic use and antibiotic resistance were extensively investigated whereas GPs’ attitudes and beliefs were less explored although considered as important determinants of prescription particularly in high prescribing countries where there can be little patient involvement [27].

The research field of culture is complex, involving overlapping disciplines such as medicine, sociology, psychology, philosophy and anthropology making a systematic approach more difficult to undertake. Free accessibility of non-medical databases was limited. Defining a level of cultural comparison was necessary and our objective was to study country-specific culture**.** Relevant papers using other terms to designate cultural differences between countries may not have been identified**.** The representativeness of countries was unequal; some countries (France, Belgium, the Netherlands and Germany) were extensively described in the publications, whereas results from other countries were limited. A possible explanation could be the contrast in antibiotic consumption motivating the choice of these countries [4]. The heterogeneous methodology of the selected papers, including both quantitative and qualitative methods, complicated quality assessment as well as extraction, interpretation and presentation of the data. We therefore decided to initially separate the results according to the methodology of the selected papers.

Limitation of the study to European countries was motivated by the researchers’ nationality, and the numerous European interventions, papers and databases concerning antibiotic use and consumption as well as resistance rates. A wider international approach could have added other aspects to the study.

Publications targeting antibiotic self-medication were included because they provided valuable cultural information about antibiotic use.

### Comparisons with published research

#### Influence of cultural dimensions on clinical practice

The findings of our literature review showed that UA (Table 3) was the most frequently quoted cultural dimension from both the practitioner’s and the patient’s perspective and proved to be significantly correlated with antibiotic consumption [12, 13]. Indeed, various authors highlight the challenge of diagnostic uncertainty faced by the practitioner or the illness outcome uncertainty motivating the patient to consult, even though the cultural perspective is not taken into account [32–35]. A French qualitative study [33] suggest that GPs use prescription to replace “uncertainty management” for which they have not been trained. The concept of uncertainty can also be considered from a wider perspective. Different attitudes towards risk-taking among GPs in different European countries were observed by Grol *et al.* [34], Dutch GPs had a lower level of “no risk-taking attitudes” than their colleagues in Belgium and the UK. Uncertainty was reported by many Belgian patients in Descheppers’ study [12] as a major motivation for rapidly seeking contact with a doctor and for requesting antibiotics. The authors describe differences in risk perception regarding disease among patients from different countries, including fear of complications of current infections and scepticism towards medicines. Gjelstad *et al.* [35] report results from the GRACE study concerning lower RTI, showing that the number of days that patients waited before consulting their GP varied from 3 to 12 between countries.

PD also influences the medical consultation (Table 2) one study showed a significant correlation with antibiotic consumption [12]. Different models of doctor-patient relationship are described in the literature and can easily be linked to levels of power distance. In a literature review, Butler *et al.* [36] describe the “paternalistic” doctor model, in which the physician knows what is best for the patient, the “patient’s choice” model after information given to the patient and the “consulting model” where information is exchanged between the doctor and the patient. Several national studies show the impact of communication between patient and practitioner on antibiotic prescribing, regardless of cultural aspects [37–40]. Meeuwesen *et al.* [41] investigated how cross-national differences in medical communication in 10 European countries can be understood through Hofstede’s cultural dimension framework. This observational study of consultations not specifically concerning antibiotics, showed that cultural norms, values, beliefs and attitudes towards health and health care may influence communication between doctors and their patients.

The third cultural dimension having proven significantly correlated to antibiotic use is MF [13] (Table 4) which is interesting to confront with patients’ differences in work ethos [27, 28] in different countries: giving priority to work in spite of illness in masculine societies or stopping work in order to allow recovery and decrease the risk of transmission of infection to others in feminine societies.

#### Interaction between education and culture

The very definition of culture is closely linked to learning and education. In our literature review, the most frequently quoted cultural determinant was patients’ attitudes, knowledge and beliefs towards infections and antibiotics (Table 1). Knowledge is a powerful determinant that can be influenced, considered either as a social factor, related to educational level [6], or as a cultural factor, depending on authors. Borg [13] suggests that knowledge can improve behaviour and thus decrease the cultural influence on antibiotic use. Radosevic *et al.* [26] found that all components of attitudes towards antibiotics were influenced by country and level of education. The 2009 Eurobarometer survey [42] suggests that persons with a higher level of knowledge take fewer antibiotics. Several information campaigns targeting the general public have been implemented in high-consuming countries such as France [43–45] and Belgium [46], contributing to increase knowledge among patients and reduce antibiotic consumption [47]. However, antibiotic consumption in both these countries remains among the highest in Europe, illustrating the challenge of educational campaigns. Another interesting European educational initiative concerning antibiotics is the e-Bug project [48] which has proved knowledge-efficient [49] initially targeting school children between 9 and 15 years of age in 18 European countries based on a country-specific needs assessment [50]. Cultural differences were taken into account when adapting and implementing it in each country [51–54]. However, educational level in general does not always exert a beneficial influence on antibiotic use. Grigoryan *et al.* [22] as well as McNulty *et al.* [55] found that one of the characteristics associated with antimicrobial self-medication was higher education.

#### Evolution of culture

Hofstede *et al.* [7] suggest that culture is a stable phenomenon exerting a very deep influence on each individual, describing a model of culture with different layers [10] (the onion diagram) where the core, represented by values, is the most difficult to change, compared to the more superficial layers or “practices”. This concept has been criticised by several authors. Classical authors, like Hegel, [56] suggest that culture undergoes continuous change. Herskovits [57] explains that culture is influenced by internal change resulting from technical or structural innovations within society (e.g. Telephone, television, internet, new laws…) and external change for example through immigration.

The World Values Surveys (WVS) [58] a global network of social scientists have conducted six surveys of values since 1981 in almost 100 countries (400 000 respondents in all) showing pervasive changes.In Blommaert *et al’s* [59] study identifying determinants of between-country differences in ambulatory antibiotic use and resistance in Europe, feelings of distrust and’feelings of religiousness (data from WVS) were the two determinants that could be considered as cultural and that were associated with total antibiotic use.

### Implications for future research

The importance of studying culture in the context of antibiotic use has often been highlighted. Grigoryan *et al.* [22] advise that strategies and campaigns to improve the situation should take cultural determinants into account. Hulsher *et al.* [32] highlight that in order to be effective, any programme to promote appropriate antibiotic use should include cultural issues and stress the importance of close international collaboration to help overcoming wide differences between countries, often due to powerful cultural factors. The context of European projects is particularly relevant to study cultural influences. Raising cultural awareness could be useful to increase the chances of success of European projects concerning antibiotic use as well as on an individual level, helping the GP to better understand patients’ expectations during the medical consultation.

Even though Hofstede’s cultural dimensions are the most frequently studied in association with antibiotic prescription, many other frameworks have been described. Narrowing the cultural research field to Hofstede’s dimensions could prove misleading. It would be safer to consider a more extensive framework for further cultural research, such as those identified by Taras *et al.* who resorted to 121 different instruments to measure culture [60].

## Conclusion

### What was already known

It has been suggested that cultural factors may influence antibiotic use.

### What this study added

This literature review identified various cultural determinants suggesting that cultural factors should not be considered as a separate category of determinants but as an ubiquitous influence on all the different stages of the disease process, from the first symptoms experienced by the patient to the decision by the practitioner to prescribe antibiotics or not, on the duration of illness and on treatment adherence by the patient. Interactions between categories of determinants, cultural dimensions and antibiotic use in primary care are multiple and complex and need further investigation which should include qualitative studies targeting both patients and GPs in order to deepen the understanding of the influence of cultural determinants on antibiotic use.

## Additional files

Additional file 1:
**Flow chart Literature review research strategy and results July 2015.** (TIF 26 kb) Additional file 2:
**Characteristics of selected articles using quantitative methods.** (PDF 35 kb) Additional file 3:
**Characteristics of selected articles using qualitative or mixed methods.** (PDF 23 kb)
