# Supplementary figures and images for: Identification of cultural determinants of antibiotic use cited in primary care in Europe: a mixed research synthesis study of integrated design “Culture is all around us”
Source: BMC Public Health. 2015 Sep 17;15:908. doi: 10.1186/s12889-015-2254-8 (PMC4574721; doi:10.1186/s12889-015-2254-8)

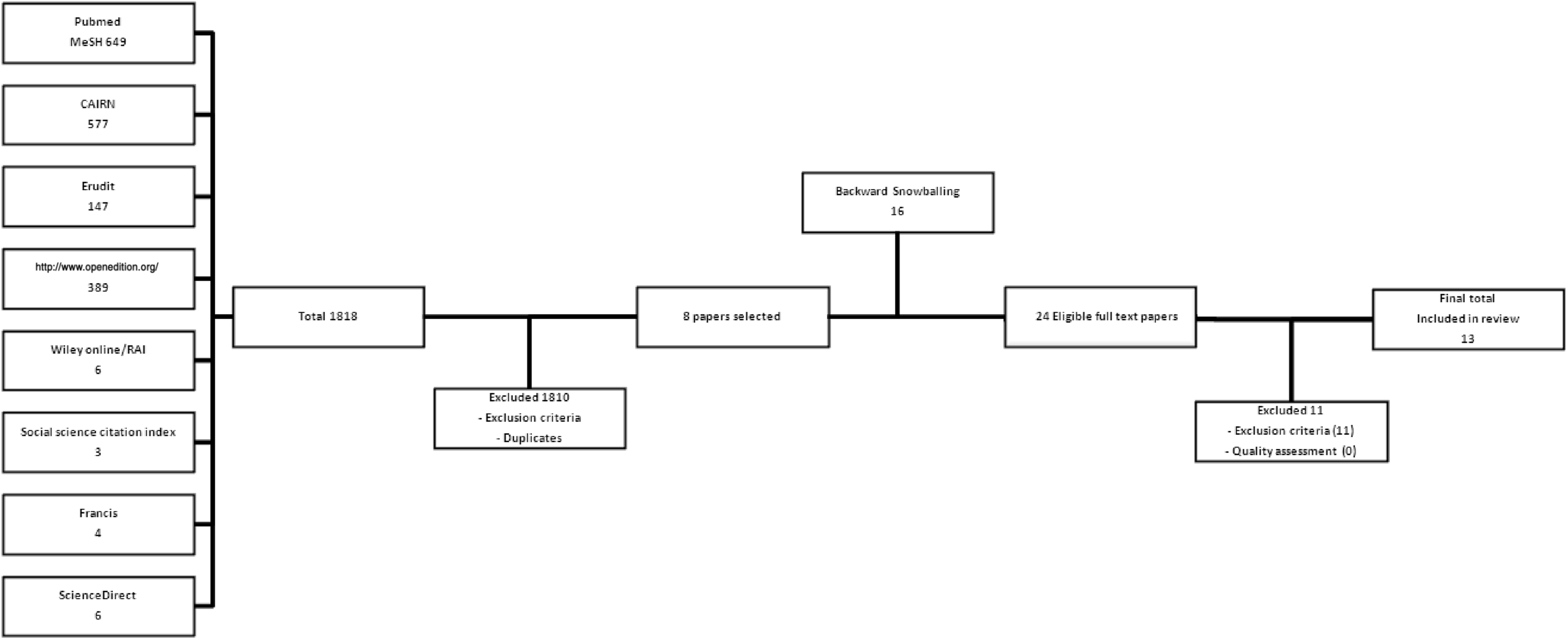

Supplement: Additional file 1: — Flow chart Literature review research strategy and results July 2015. (TIF 26 kb) [file 12889_2015_2254_MOESM1_ESM.tif]
