# Supplementary material for: Identification of cultural determinants of antibiotic use cited in primary care in Europe: a mixed research synthesis study of integrated design “Culture is all around us”
Source: BMC Public Health. 2015 Sep 17;15:908. doi: 10.1186/s12889-015-2254-8 (PMC4574721; doi:10.1186/s12889-015-2254-8)
Supplement: Additional file 2: — Characteristics of selected articles using quantitative methods. (PDF 35 kb) [file 12889_2015_2254_MOESM2_ESM.pdf]

Additional file 2 : Characteristics of selected articles using quantitative methods					
**First author, year of publication**	**QA score**	**Method of data collection**	**Number of participating countries**	**Sampling**	**Main results**
de Melker 1997 [18]	EPHPP 2	Questionnaire targeting acute tonsillitis treated by antibiotics.	7 European countries	2423 patients through their GP	Clinical outcome: Initial temperature and duration did not show much variation according to country<br>Illness-related behaviour, number of days of illness prior to presentation to the GP and duration of illness varied according to country
Pechèrel 2001[19]	EPHPP 2	Questionnaire (by telephone) targeting perceptions of RTI and attitudes towards antibiotic use.	9 countries: (5 European and 5 international)	5379 randomized adult patients	Patients' compliance and attitudes towards antibiotics and respect for practitioners varied according to the country:<br>Hypotheses put forward to explain these differences: Access to medical care facilities, health insurance system, education, cultural background.
Pechère 2002 [20]	NICE ++	Questionnaire (face-to-face) targeting perceptions of antibiotics, influence of doctor's attitudes and behaviour in the management of respiratory tract infections (RTI).	4 European countries	3200 RTI adult patients	Definition of 4 patient types according to attitudes towards the practitioner which varied across participating countries: 2 positive attitudes towards the doctor: Involved patients (mainly from Germany and France) and deferent (mostly from France and Spain) with more trust in their practitioner and 2 negative attitudes: Ignored patients (mostly in Italy) and critical (mostly in France and Italy)
van Duijn 2003 [21]	EPHPP 2	Questionnaire targeting patients' views on RTI symptoms and antibiotics.	3 European countries	678 randomized adult patients	Patients' attitudes towards RTI symptoms varied in each country: Belgian patients considered RTI symptoms more serious and less self-limiting than Dutch and UK patients and reported a greater need to consult a GP .<br>Patients' attitudes towards antibiotics (effectiveness to speed recovery and prevent complications) were similar in all 3 countries .
Grigoryan 2006 [22]	EPHPP 2<br>NICE ++	Questionnaire targeting self-medication with antibiotics.	19 European countries	15548 randomized adult patients	A North-South and East-West gradient in antibiotic self-medication was observed: Southern and Eastern countries were more likely to practice self-medication than Northern and Western European countries.
Grigoryan 2007 [23]	EPHPP 2<br>NICE ++	Questionnaire (face-to-face) targeting patients' attitudes, beliefs and knowledge towards antibiotic use and self-medication.	12 European countries	1200 patients	Respondents from Southern and Eastern countries had less appropriate attitudes, beliefs, knowledge and awareness of antibiotic resistance compared to North-Western countries,
Rosman 2008 [24]	EPHPP 2	Data from GP data basis SFMG* and LINH** targeting diagnostic and antibiotic prescription patterns for RTI management in primary care	2 European countries	268 GP practices	The number of consultations for URTI was higher in France.<br>No difference in the volume of antibiotic prescriptions was observed.<br>Prescriptions for symptomatic treatments were more frequent in France.<br>Hypotheses put forward by the authors: cultural differences linked to patient's health seeking behaviour and medication perception.

Grigoryan 2008 [25]	EPHPP 2 NICE++	Questionnaire (face-to-face) face-to-face targeting patients' attitudes, beliefs and knowledge towards antibiotic use and self-medication.	12 European countries	1200 patients	The observed predisposing factors for self medication with antibiotics were high perceived appropriateness of self-medication with antibiotics for bronchitis and an attitude favouring antibiotic use for minor infections.
Deschepper 2008 [**Erreur ! Signet non défini.**]	EPHPP 2 NICE ++	Country-level data from 3 different European databases targeting antibiotic consumption; ESAC†, SAR††and the Eurobarometer 2002 were correlated to country-specific scores for Hofstedes' cultural dimensions.	27 European Countries	SAR:15548 randomized adult patients Eurobarometer 16230 randomized ≥ 15 year olds	Two main cultural dimensions were significantly associated with antibiotic consumption: Antibiotic consumption increased with a high score of Power distance (all 3 databases) and Uncertainty avoidance(2 of the 3 databases):<br>After controlling for wealth (Gross Domestic Product, GDP) these correlations were not confirmed whereas antibiotic consumption increased with masculinity score.
Radosević 2009 [26]	EPHPP 2	Questionnaires exploring patients' cognitive, affective and behavioural components regarding antibiotics.	6 European countries	838 adult participants in a convenience sampling	All components of attitudes towards antibiotics were influenced by national differences and characteristics as well as level of education. Respondents from Slovenia showed the best knowledge about antibiotics while the lowest was observed in Fyrom. The most positive emotions about antibiotics were expressed in Greece and Hungary and the most negative in Slovenia.
Borg 2011 [**Erreur ! Signet non défini.**]	EPHPP 2 NICE ++	Data from the 2009 Eurobarometer survey were extracted targeting antibiotic consumption for viral infections, appropriate antibiotic use in the previous year, knowledge about antibiotics and were correlated to Hofstede's cultural dimensions.	27 European countries	26259 randomized ≥ 15 year olds	2 cultural dimensions were significantly correlated to antibiotic consumption:, Antibiotic consumption increased with a high score of Uncertainty avoidance, and Masculinity versus Femininity.

*Société Française de Médecine Générale
**Dutch Landelijk Informatie Netwerk Huisatsenzorg
†European surveillance of antibiotic consumption
†† Self-Medication with Antibiotics and Resistance in Europe
