# Supplementary material for: Identification of cultural determinants of antibiotic use cited in primary care in Europe: a mixed research synthesis study of integrated design “Culture is all around us”
Source: BMC Public Health. 2015 Sep 17;15:908. doi: 10.1186/s12889-015-2254-8 (PMC4574721; doi:10.1186/s12889-015-2254-8)
Supplement: Additional file 3: — Characteristics of selected articles using qualitative or mixed methods. (PDF 23 kb) [file 12889_2015_2254_MOESM3_ESM.pdf]

Additional file 3: Characteristics of the selected articles using qualitative or mixed methods

Reference	QA score	Method	Country	Sampling	Main results
Deschepper et al 2002 [27]	NICE ++	Semi-structured individual interviews before and after completion of a 3 months diary targeting RTI.	2 European countries	30 purposefully selected patients	Several categories of differences between the countries were identified:<br>Disease labelling : Belgian patients mostly used the label « bronchitis » whereas Dutch patients, mostly used «cold» or"flu". Initial coping actions which were closely linked to the labelling of disease:.« bronchitis » was followed by a consultation with the GP whereas «cold» and "flu". were followed by symptomatic self-medication and nursing one's illness.<br>Antibiotic use: Antibiotics were mainly used for conditions labelled as «bronchitis» or RTI with complications by the Belgian sample and only for the latter by the few Dutch patients treated with antibiotics<br>Patients' expectations: Belgian patients expect their doctor to prescribe, more than Dutch patients.<br>Patients' attitudes towards antibiotics Four different categories of attitudes towards antibiotics were distinguished: "Better safe than sorry" where people preferred not to wait too long before taking antibiotics (only Belgian patients). "Antibiotics, if there is no alternative" where people showed strong aversion to antibiotic use even though they used quite a lot of antibiotics and passed on the responsibility to the doctor (mostly Belgian patients). «Rather not, but accepting» where people had a positive image of antibiotics and left the decision to the doctor in trust (mostly Dutch patients). "Refusal" where people refused antibiotic use. (both Belgian and Dutch patients).<br>Risk perception: Uncertainty was reported by many Belgian patients and was an important factor for quickly seeking contact with a doctor and demanding antibiotics. Dutch patients did not believe that either the doctor or the antibiotics had much impact on the course of the episode.<br><br>.

Rosman 2009 [28]	NICE ++	Semi-structured individual interviews with general practitioners and observation of consultations in GP practices.	2 European countries	53 interviews, and 150 observed consultations Purposeful sampling.	Several categories of differences and similarities were observed:<br>All GPs from both countries agreed that antibiotics were the prescriptions that induced the most negotiations with their patients.<br>However the prescription context is different in each country:<br>Initial medical training: the training of French GPs was described as more hospital-centred than in the Netherlands, encouraging frequent prescribing driven by fear of complications. In the Netherlands GPs were trained to follow the general practice guidelines and training was centred on pathologies encountered in outpatient care.<br>Legal complaints: fear of legal issues can lead to antibiotic prescription.<br>Retribution system: the fee-for-service system in France results in a commercial transaction with patients and competition between GPs.<br>Practice context: Dutch GPs have been reducing prescriptions over several decades, GP consultations being centred on patient education strategies, whereas for French GPs this prescription behaviour is new and requires a change of practice as well as a change in patients' expectations, sustained by previous prescriptions and misunderstandings.
